# Supplementary material for: Patterns of Intron Gain and Loss in Fungi
Source: PLoS Biol. 2004 Nov 30;2(12):e422. doi: 10.1371/journal.pbio.0020422 (PMC532390; doi:10.1371/journal.pbio.0020422)
Supplement: Table S1 — Also available at http://genes.mit.edu/NielsenEtAl/. (4.3 MB ZIP). [file pbio.0020422.st001.zip › NielsenEtAl/html/1204.html]

AN8232.1.NCU07757.1.MG04877.1.FG09011.1


```
 CLUSTAL W (1.82) Multiple Sequence Alignments - Introns Inserted


Sequence 1: NCU07757.1	465 aa
Sequence 2: FG09011.1	463 aa
Sequence 3: MG04877.1	511 aa
Sequence 4: AN8232.1	445 aa
Alignment Length: 512 aa
Number Identitical Residues: 190 aa
Alignment Score (without introns) 10329


MG04877.1 	MASAPAAAATAGAGQAASGAAARVEDNAPKLLWNTENVKDVAESVGIPNINDDAVRCLAQ
NCU07757.1	MAGE-----------------------GPKLLWNPENVKDVAESIGIN-LTEEPLRVLTQ
FG09011.1 	MAAE-----------------------TPKLLWNPDNVKDVAESVGISSLNEEALKCLTQ
AN8232.1  	-----------------------------MSVWNPDNIRDVAESVGIVNLNNDVTENLAR
          	                               :**.:*::*****:** .:.::  . *::

MG04877.1 	DVEYRIGQVLVEALRFMRAANRTTLTVQDTSQAFKVLDVEPLYGYDSTRPLRYGEASLGP
NCU07757.1	DVEYRIGQVVVEALRFMRAANRTTLTVQDVSQALRVLDVEPLYGYDSTRPLRYGEASIGP
FG09011.1 	DVEYRVGQVIVEALRFMRASRRTTLTVNDISLALRALDVEPLYGYDSTRPLRYGEASLGP
AN8232.1  	DVEYRIAQVLEEALKFMRHSRRTLLTTQDIALALRVLDVEPLYGYETTRPLKFGEASLGP
          	*****:.**: ***:*** :.** **.:* : *::.*********::****::****:**

MG04877.1 	GQPLFYIDDEEVDFEKLINAPLPKVPRDMSFTA1HWLAVEGVQPSIPQNPTTAESRAQEL
NCU07757.1	GQPLFYIEDEEVDFEKVINAPLPKVPRDMSFTA1HWLAIDGVQPSIPQNPTTAETSSKDL
FG09011.1 	GQPLFYIEDEEVDFEKLINAPLPKVPRDMGFTA1HWLAIEGVQPSIPQNPTTSESRSQEL
AN8232.1  	GQPLFYVEDEEVDFEKLINAPLPRVPREISFTA1HWLAVEGVQPSIPQNPTAADSRNLEL
          	******::********:******:***::.*** ****::***********::::   :*

MG04877.1 	IPKGPGANPALNALAGNDNLAFHPAVKQIISKELVLYFDKIQAAVLDDTPDEEVVRLRNA
NCU07757.1	LPKGPGANPAVAALAGNDNVAFRPAVKHVISKELILYFDKVQAAIMDDDPDEEKTRLRSA
FG09011.1 	LPKGPGANPALSALAGNDNVSMKPSVKHIVSKELILYFDKIQAAVLDDNPDEEVVRLRQA
AN8232.1  	MSKGPNANATLAAMSGNGNVAVKPLVKHVLSKELQLYFEKVCNAFLDESSE----KYRTS
          	:.***.**.:: *::**.*::.:* **:::**** ***:*:  *.:*: .:    : * :

MG04877.1 	ALASVRNDPGLHQLVPYFANFIANQVTHRLDDTFTVRQMMELTAALVDNSNLFLDPY~AG
NCU07757.1	ALDSVRSDPGLHQLVPYFVSFINNQVTHRLDDLFVLRQMMELTGAILDNPSIFLDPY~AS
FG09011.1 	ALGSVRDDPGLHQLIPYFITFIMDRVTHHLDDTFTLKQMMELTNALIENKSLFLDPY~AS
AN8232.1  	GYASLREDPGLHQLVPYFVQFISEKVTHGLKDIFVLTQVMHMAEALVQNKSLYVDPY0IA
          	.  *:*.*******:***  ** ::*** *.* *.: *:*.:: *:::* .:::***  .

MG04877.1 	PLSAPVLTALMSRKIGASTSTSKQEEGSGSGNSSSVNREALREQFLLREFAASLLGKIAI
NCU07757.1	SLAAPVLTCLMARKLGGSES---SLEGT----------DALKDQYRLREVAASLLGTIAR
FG09011.1 	PLSAPALTCLMARKLG-------TEDGT----------DALKEQYELRQLAASLVGQIAR
AN8232.1  	SLVPPILTCLIGRQLG----------GT----------ADLSEQFALRDLAAALLGLIAT
          	.* .* **.*:.*::*          *:            * :*: **:.**:*:* ** 

MG04877.1 	KYTRANRHLRSKLTRTCLKYLLDPTKPAPVLFGALNGLAAAGGPEAFRILVLPNLREFDT
NCU07757.1	KYSKTNALLRPKLTRTCLKFFLDPSKSPAVLYGAISGLAAAGGPEAVRILVLPNLKMFDE
FG09011.1 	KYSASNTLLRPKLTRTCLKYFLDPTKPPAVLYGAIHGLLEAGGPEAIRVLVLRNLKTFDS
AN8232.1  	KYSHSSHTLKPRLARSCLKTLLDPSKPFGAHYGAIIGLHAVGGSEAVRVLIIPNLPIYGN
          	**: :.  *:.:*:*:*** :***:*.  . :**: **  .**.**.*:*:: **  :. 

MG04877.1 	GMLQPLQEKAANAAGAAGTVAQIEFEALVGAIVKAIMSLVEHDPAVPNGVNGGTS-ESES
NCU07757.1	GILTPLREKGEASH--------FEYEALVGGIMKAIETLVEGITLPV--VADVAELEREG
FG09011.1 	GILQPLREKMEGS---------MDYEMLVQGIVQAVASLADHISSDTNGINGSTSVEGEA
AN8232.1  	LLKDGLADDSARRP---------EAEKVLGLLLGILTALKEEKPVLTNGHGPGMVSEDIK
          	 :   * :.              : * ::  ::  : :* :  .   ..       *   

MG04877.1 	AELVEFLGPIVGRRIADKGDHKLVRVVLAARNIE
NCU07757.1	QLVIEFLGRTIGERVARLGNQHLNRGILEVRHLE
FG09011.1 	SELKDFIGPIIGGKIVASGNRRLIRTILDARNLE
AN8232.1  	RRLEGKLGAFVAEKVAELGDARVAHAILG-----
          	  :   :*  :. ::.  *: :: : :*
```
